# Differences in gluten protein composition between old and modern durum wheat genotypes in relation to 20th century breeding in Italy

**DOI:** 10.1016/j.eja.2017.04.003

**Published:** 2017-07

**Authors:** Michele A. De Santis, Marcella M. Giuliani, Luigia Giuzio, Pasquale De Vita, Alison Lovegrove, Peter R. Shewry, Zina Flagella

**Affiliations:** a Dipartimento di Scienze Agrarie, degli Alimenti e dell’Ambiente, Università degli Studi di Foggia, Via Napoli 25 - 71122, Foggia, Italy; b Consiglio per la Ricerca in Agricoltura e l’Analisi dell’Economia Agraria – Centro di Ricerca per la Cerealicoltura (CREA-CER), S.S. 673 km 25.200, 71122 Foggia, Italy; c Department of Plant Biology and Crop Science, Rothamsted Research, Harpenden, Hertfordshire AL5 2JQ, UK

**Keywords:** Durum wheat old and modern genotypes, Gluten, Wheat allergy, ω-gliadin, Sds-page

## Abstract

The impact of breeding on grain yields of wheat varieties released during the 20th century has been extensively studied, whereas less information is available on the changes in gluten quality associated with effects on the amount and composition of glutenins and gliadins. In order to explore the effects of breeding during the 20th century on gluten quality of durum wheat for processing and health we have compared a set of old and modern Italian genotypes grown under Mediterranean conditions. The better technological performance observed for the modern varieties was found to be due not only to the introgression of superior alleles of high (HMW-GS) and low molecular weight (LMW-GS) glutenin subunits encoded at *Glu-B1* and *Glu-B3 loci*, but also to differential expression of specific storage proteins. In particular, the higher gluten index observed in modern genotypes was correlated with an increased glutenin/gliadin ratio and the expression of B-type LMW-GS which was, on average, two times higher in the modern than in the old group of durum wheat genotypes. By contrast, no significant differences were found between old and modern durum wheat genotypes in relation to the expression of α-type and γ-type gliadins which are major fractions that trigger coeliac disease (CD) in susceptible individuals. Furthermore, a drastic decrease was observed in the expression of ω-type gliadins in the modern genotypes, mainly ω-5 gliadin (also known as Tri a 19) which is a major allergen in wheat dependent exercise induced anaphylaxis (WDEIA). Immunological and 2DE SDS-PAGE analyses indicated that these differences could be related either to a general down-regulation or to differences in numbers of isoforms. Lower rainfall during grain filling period was related to overall higher expression of HMW-GS and ω-gliadins. In conclusion, breeding activity carried out in Italy during the 20th century appears to have improved durum wheat gluten quality, both in relation to technological performance and allergenic potential.

## Highlights

• Higher B-type LMW-GS expression in modern genotypes contributes to gluten strength.
• A marked decrease in ω-type gliadin expression is observed in modern genotypes.
• Differences in ω-5 gliadin expression appear also related to the number of isoforms.
• Old and modern genotypes show similar α- and γ-type gliadin expression.
• A higher ω-gliadins and HMW-GS content was observed under water deficit.

## Graphical abstract

**Figure:**
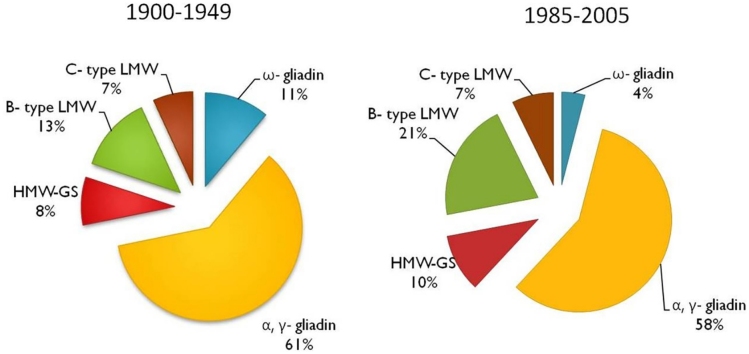

## 1 Introduction

Durum wheat (*Triticum turgidum* L. spp. *durum*) is a worldwide crop, cultivated on about 30–35 million hectares and in particular in the Mediterranean basin where it represents a staple crop (Guzmán et al., 2016). Endosperm prolamins, accounting for up to 80% of the total grain proteins, determine the technological quality of durum wheat flour (semolina) that is mostly used for the production of pasta, bread and cous cous. These alcohol-soluble grain storage proteins are classified, based on their electrophoretic mobility, into monomeric gliadins (α-, γ- and ω-) and polymeric glutenins (comprising high and low molecular weight glutenin subunits, HMW-GS and LMW-GS). HMW-GSs are subdivided into x- and y-types, encoded by closely linked genes at the *Glu-A1* and *Glu-B1* loci on the long arms of chromosomes 1A and1B in durum and other tetraploid wheats (which lack the D genome and associated storage proteins present in bread wheat). The LMW-GSs are classified into B-, C- and D-subunits, according to their structural and functional properties. On the basis of N terminal amino acid sequences, three subgroups of typical LMW-GS can be recognized, called LMW-s, LMW-m and LMW-i types, according to the first amino acid residue of the mature protein: serine, methionine or isoleucine, respectively (D’Ovidio and Masci, 2004). They are encoded on the chromosomes 1 at the *Glu-A3* and *Glu-B3* loci (1A and 1B) and at the loci tightly linked to the *Gli-1* and on the group 6 chromosomes at *Gli-2* loci. The B-subunits are typical LMW-GS with a characteristic structure and are encoded by genes on the group 1 chromosomes while the C- and D- subunits are gliadin-like proteins encoded by genes on the group 6 chromosomes. Although these proteins are structurally similar to gliadins they act functionally as glutenins, due to their ability to form intermolecular disulfide bonds between unpaired cysteine residues. The γ-type gliadin subunits are encoded on the short arms of group 1 chromosomes by genes at the *Gli-1* loci, while the α- and β- subunits are encoded by genes at the *Gli-2* loci on the short arms of the group 6 chromosomes. In durum wheat ω-type gliadins are encoded on the short arms of group 1 of chromosomes A and B, with *Gli-B1* termed as ω-5 gliadin and Gli-A1 as ω-1 gliadin. They are also distinguished by their N-terminal amino acid sequences,SRLLSPQ in ω-5 gliadins, ARQLNPSNKELQ or KELQSPQQS in ω-1 gliadins (Wan et al., 2014).

It is well documented that pasta-making quality is related to the gluten protein composition (Peǹa et al., 1994, Edwards et al., 2007, Sissons, 2008), which can be influenced either by genetic (De Vita et al., 2007, Pompa et al., 2013, Subira et al., 2014) or environmental factors (Triboi et al., 2000, Altenbach, 2012, Giuliani et al., 2014, Giuliani et al., 2015). Polymorphism in HMW and LMW glutenin subunits is associated with differences in dough quality. Several studies of bread and durum wheats have demonstrated that the HMW-GS 7 + 8 alleles are associated with better quality compared with the allelic HMW-GS 20 (De Vita et al., 2007, Nazco et al., 2014). This may relate to differences in protein amount and structure (Shewry et al., 2003, Cunsolo et al., 2004). The LMW-GS, especially subunits encoded by loci on chromosome 1B (*Glu-B3*), also affect the end-use quality of durum wheat; in particular, the specific LMW-2 group of subunits is associated with the best pasta-making properties while the LMW-1 group is associated with poor pasta-making characteristics (Peǹa et al., 1994, D’Ovidio and Masci, 2004). During the 20th century, durum wheat breeding aimed at releasing cultivars with higher grain yield, shorter stature and early maturation. The introgression of the gene for the reduction of the height (Rht) from a series of CIMMYT lines (Norin 10 – *Triticum aestivum* L.) resulted in the introduction of genotypes with higher potential yield and reduced risk of lodging (De Vita et al., 2007).

During the last half of the past century one of the main goals of breeding programs in Mediterranean environments was the release of durum wheat cultivars with high quality standards (Subira et al., 2014). An increase in pasta-making quality during the 20th century in durum wheat cultivars released in Italy was reported by De Vita et al. (2007) due to the incorporation of favorable gluten protein alleles in modern cultivars, such as the HMW-GS 7 + 8 allele encoded by the *Glu*-*B1* locus. Subira et al. (2014) investigated the genetic improvement of quality traits of durum wheat achieved in Italy and Spain during the 20th century by using an historical series of 12 cultivars from each country in order to determine the relationship between allelic variation in HMW-GS and LMW-GS composition and gluten strength which increased by 32.1% (0.54% year^−1^) and 27.9% (0.33% year^−1^), respectively, in Italian and in Spanish cultivars. A much stronger genetic control of gluten strength was found in Italian than in Spanish cultivars. The improvement in gluten quality was also related to the progressive incorporation into recent cultivars of favorable LMW-GS. Breeding was found to increase the frequency of the LMW combination *aaa* (allele 6 at *Glu-A3*, alleles 2 + 4 + 15 + 19 at *Glu-B3*, allele 12 at *Glu-B2*), which was present in 75% of all intermediate cultivars and in 100% of the modern Italian cultivars. Breeding programs were also successful in increasing the stability of gluten strength and the sedimentation index. The contribution of gliadins to viscosity and extensibility is also well documented (Shewry, 2009), although not particularly relevant in pasta-making quality.

There is also an increasing interest in the characterization of gluten proteins due to the immune stimulating effects of certain sub-fractions (mostly gliadins) on susceptible human patients. Wheat allergy (WA) and coeliac disease (CD) are currently considered the main immune-mediated disorders associated with the ingestion of gluten in diet (Sapone et al., 2012). The list of the known wheat allergens includes both soluble albumins and globulins and prolamin storage proteins (Matsuo et al., 2015). Among the wheat allergens, ω-5 gliadin (or Tri a 19) is known to be the major protein responsible for wheat-dependent exercise-induced anaphylaxis (WDEIA; Matsuo et al., 2005). Currently thirty-one amino acid peptide sequences in the prolamins of wheat and related species have been defined as epitopes capable of inducing a response in susceptible individuals with coeliac disease (Sollid et al., 2012, Shewry and Tatham, 2016). However, the mapping of coeliac epitopes is incomplete and the number of distinct epitopes a matter of on-going discussion (Sollid et al., 2012). Analyses of hexaploid wheat, including modern genotypes and local landraces, also showed genetic differences in the relative amounts of the α9- and α20- epitopes involved in CD (van den Broeck et al., 2010, Salentijn et al., 2013). Recently Ribeiro et al. (2016) reported that breeding does not appear to have contributed to the prevalence of potential coeliac disease immune-stimulatory epitopes.

While the influence of breeding on the polymorphism of durum wheat glutenin and gliadin subunits has been extensively investigated, very few studies have been reported about differences in the level of expression of gluten protein sub-fractions in relation to both technological and health properties.

In order to explore the changes in gluten composition and quality resulting from breeding of Italian durum wheats in the 20th century, we have compared eight modern and seven old Italian durum wheat genotypes grown in a two year field trial. The gluten protein composition has been determined by SDS-PAGE to identify differences in gliadin and glutenin subunit composition and expression with particular attention to differences in ω-5 gliadin expression.

## 2 Materials and methods

### 2.1 Plant material

Fifteen durum wheat (*Triticum turgidum* spp. *durum*) genotypes were chosen based on the year of release from 1900 to 2005 and subdivided into two groups (old and modern), as showed in Table 1.&lt;!--OT##Please note the author comments---"In Table 1, please GluA1 and GluB3 columns shall be elnarged, since both titles and parameters would fit in one row. In Tables 2 and 3, in the first row, each parameter (Yield, TKW etc..) should be centered for each couple of years column (2013 and 2014). In Table 4 parameter titles must be included in a single row (to not go head to head); it would be better to enlarge each column."--&gt; The old genotypes group includes seven entries commonly released in Italy from 1900 until 1949 and the modern one contains eight cultivars released after 1985 and carrying the *Rht* genes. Pedigree, phenological characteristics and allelic differences in glutenin composition (HMW and LMW) are reported in Table 1. Most of the genotypes had the *Glu-B1* HMW-GS 7 + 8 and *Glu-A1* null alleles (8/15) and the *Glu-B3* allele LMW-2 (14/15).

**Table 1:** List of the investigated genotypes with some genetic details.

Groups	Genotypes	Pedigree	Year of release	Earliness*	Glu A1	Glu B1	Glu B3
Old	Dauno III	landraces from south Italy	1900	34	2	6 + 8	LMW-2
	old Saragolla	landraces from south Italy	1900	30	null	7	LMW-2
	Russello	landraces from Sicily, Italy	1910	30	null	13 + 16	LMW-1
	Timilia (R.B.) “reste bianche”	landraces from Sicily, Italy	1910	29	null	20	LMW-2
	Cappelli	selection from Tunisian population Jean Retifah	1915	31	null	20	LMW-2
	Garigliano	Tripolino × Cappelli	1927	28	null	7 + 8	LMW-2
	Grifoni 235	Cappelli* × Triticum aestivum*	1949	28	null	7 + 8	LMW-2

Modern	Adamello	Valforte × turkish line 7116	1985	23	null	7 + 8	LMW-2
	Simeto	Capeiti 8 × Valnova	1988	22	null	7 + 8	LMW-2
	Preco	(Edmore × WPB881) × Selected line 3	1995	22	null	6 + 8	LMW-2
	Iride	Altar 84 × Ionio	1996	24	null	7 + 8	LMW-2
	Svevo	Cimmyt line × Zenit	1996	24	null	7 + 8	LMW-2
	Claudio	(Cimmyt selection × Durango) × (IS1938 × Grazia)	1998	25	null	7 + 8	LMW-2
	Saragolla	Iride × PSB 014 line	2004	23	null	6 + 8	LMW-2
	PR22D89	(Ofanto × Duilio) × Ixos	2005	26	null	7 + 8	LMW-2

* (heading date expressed in number of days from April 1st).

### 2.2 Field trials

Plants were grown in the field on a clay–loam soil (Typic Chromoxerert) at Foggia (Italy, 41° 28′ N, 15° 32′ E and 75 m a.s.l.), in two consecutive growing-seasons (2012–2013 and 2013–2014, indicated below as 2013 and 2014, respectively). The average main physical and chemical soil characteristics of the two years were: 36% clay, 17% silt and 47% sand; pH 7.8; 17.3 g kg^−1^ organic C; 1.5 g kg^−1^ total N; 19 mg kg^−1^ available P; 111 mg kg^−1^ exchangeable K. The previous crop was durum wheat. Field trials were carried out at Consiglio per la Ricerca in agricoltura e l'analisi dell' Economia Agraria, Centro di Ricerca per la Cerealicoltura (CREA-CER − Foggia, Italy). A randomized complete block design with three replications was adopted. Seeds came from gene bank of CREA-CER. Seeding density was 350 seed m^−2^. Sowing time was in the first 10 days of December of both crop seasons. Plots consisted of 8 rows, 7.5 m long, corresponding to an area of about 10 m^2^. In both years nitrogen and phosphorous were applied at a rate of 80 kg ha^−1^ and 70 kg ha^−1^, respectively. A low nitrogen (N) input was used according to the standard agronomic practices adopted in the Mediterranean area, in particular to reduce lodging in old genotypes. Nitrogen fertilizer was applied at two stages: 1/3 at sowing date (150 kg ha^−1^ of bi-ammonium phosphate N 18%, P 46%) and 2/3 at tillering stage (200 kg ha^−1^ of ammonium nitrate N 26–27%). Weeds within the growing seasons were controlled with the following herbicides: Tralcossidim (1.7 L ha^−1^) + Clopiralid + MCPA + Fluroxypyr (2.0–2.5 L ha^−1^). Whole plots were harvested mechanically early in June each year and grain yield determined at 13% moisture content (t ha^−1^). Heading date was recorded when about half of the culms showed emerging spikes (growth stage 55; Zadoks et al., 1974). Varietal earliness was expressed as heading date, as shown in Table 1.

### 2.3 Weather conditions

**Fig. 1:**
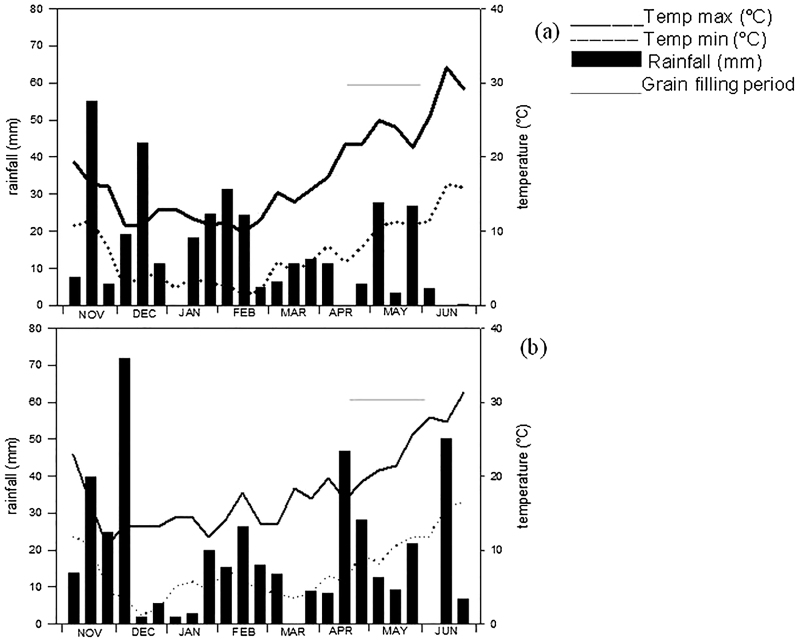
Rainfall distribution and ten-day maximum and minimum mean temperatures in the 2012/13 (a) and 2013/14 (b) crop seasons.

Rainfall distribution and maximum and minimum ten-day period mean temperatures during the 2013 (a) and 2014 (b) crop seasons are reported in Fig. 1. With the exception of December and January, higher and more evenly distributed rainfall occurred in the second crop season compared with the first. This trend was more evident during grain filling when the amount of rainfall that occurred from the 2nd ten-day period of April (flowering) to the 2nd ten-day period of June (harvest) was three times higher in the second crop season (153 vs 54 mm). By contrast, the trends for maximum and minimum temperatures were similar between the two years.

### 2.4 Yield and grain quality

At harvest, the grain yield (t ha^−1^) and its main components, 1000 kernel weight (TKW) and number of kernels m^−2^, were determined. Several commercial and technological quality parameters were also determined. Test weight, moisture and grain protein content were determined by NIR (Infratec 1241 Analyzer, Foss, Hillerod, Denmark). Semolina flour was obtained from kernels milled by Bona mill 4 cylinders (sieve 180 μm). The gluten index (GI), an indicator of the gluten strength, was determined using the Glutomatic system (Perten Instruments, Sweden) according to ICC standard 155 (International Association for Cereal Science and Technology, 1986).

### 2.5 Gluten protein extraction and SDS-PAGE

**Fig. 2:**
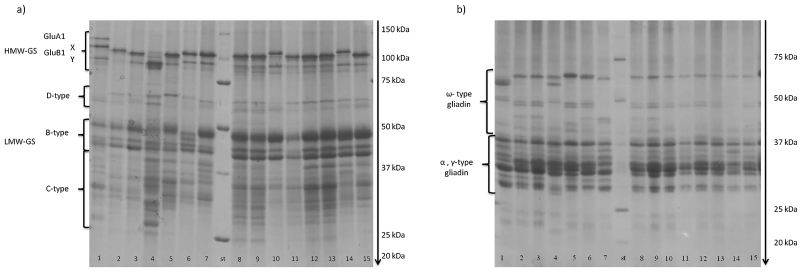
SDS-PAGE of glutenins (a) and gliadins (b). Samples were ordered according to the release date (from left to right): 1) Dauno III; 2) old Saragolla; 3) Cappelli; 4) Russello; 5) Timilia RB; 6) Garigliano; 7) Grifoni 235; 8) Adamello; 9) Simeto; 10) Preco; 11) Svevo; 12) Claudio; 13) Iride; 14) Saragolla; 15) PR22D89; st) molecular weight ladder.

Endosperm storage proteins were extracted according to Hurkman and Tanaka (2004) modified according to Singh et al. (1991). Briefly, 100 mg of flour was suspended in 0.4 mL of KCl buffer (pH 7.8) and centrifuged to remove soluble proteins. The KCl-insoluble fraction was suspended in 1-propanol solution (50% v/v) and centrifuged for 10 min at 4500 *g* (repeated twice) to separate gliadins from glutenins. The protein content of the extracted gliadin and glutenin fractions was measured by the Biuret method. Extracted gliadins and glutenins (10 μL) were separated by SDS-PAGE (T 12%, C 1.28%), as shown in Fig. 2, at 25 mA per gel for 4 h at 10 °C, using an SE 600 apparatus (Hoefer, Inc., Holliston, MA, USA). Gels were stained with Coomassie Brilliant Blue G250 according to Neuhoff et al. (1988), distained with tap water, scanned at 300 dpi (Epson Perfection V750pro) and analysed by software ImageQuant Tl (GE Healthcare Bio-Sciences AB). Three biological and three technical replicates were analysed. Protein molecular weight marker (10–250 kDa) was used (Precision Plus Protein™ Unstained Standards, Bio-Rad). Band intensity was quantified as total and relative volumes, after removing background. Due to the well-known overlap of some γ-type in the α-type gliadin gel region (Tatham and Shewry, 1985), gliadins were subdivided into two classes (ω- and α-, γ-) on the basis of their molecular weight according to Tosi et al. (2011) while glutenins were subdivided into HMW-GS and LMW-GS (B- and C-type) according to Shewry and Tatham (2016). In order to compare the storage protein composition, the amounts of sub-fractions were calculated relative to the total extracted storage proteins. The ratio of gliadin on glutenin (*glia:glut*) was determined as the ratio of the amounts of extracted gliadins and glutenins. The ratio of HMW-GS to B-type LMW-GS was also determined. In addition, the storage protein compositions of the two groups of genotypes were compared after normalization for the flour protein content, to avoid differences relating to differences in grain protein contents between the two groups.

### 2.6 Analysis of ω-5 gliadin expression

Five old (Dauno III, old Saragolla, Cappelli, Russello, Timilia RB) and four modern (Claudio, Iride, Saragolla and PR22D89) genotypes were analysed by western blot based on ω-gliadin polymorphism. A monoclonal primary antibody (Ab) raised against the N-terminal sequence (SRLLSPRGKELGC) of ω-5 gliadin (Denery-Papini et al., 1994, Denery-Papini et al., 2000) was used according to the protocol reported by Wan et al. (2014). Following the outcome of western blot, the proportion of the ω-5 gliadin on the total ω-type gliadin expression evaluated by gel image analysis, was determined. Furthermore, on the basis of the results of the western blotting, gliadins from one old (Dauno III) and one modern (Saragolla) genotype were analysed by 2D SDS-PAGE, according to Giuliani et al. (2015). Briefly, 200 μg of extracted gliadins were separated by iso-electro focusing (IEF) using Immobiline DryStrips (pH 3–10, 13 cm length; GE Healthcare Bio-Sciences AB; IEF volt conditions were: 12 h of rehydration at 20 °C, +1 h 500 V, +1 h 1000 V, +2 h 5000 V, +4 h 8000 V, 30 mA per gel, 28000–38000 total volt hrs). SDS-PAGE was then carried out using an SE 600 apparatus (Hoefer, Inc., Holliston, MA, USA). Strips were reduced with DTT (only glutenin), alkylated by iodoacetamide (IAA), equilibrated and then loaded onto 1 mm × 18 cm × 16 cm vertical polyacrylamide SDS-PAGE gels (T, 12%; C, 1.28). Image analysis was performed using Image Master 2D Platinum 6.0 (GE Healthcare Bio-Sciences AB).

### 2.7 Statistical analysis

**Fig. 3:**
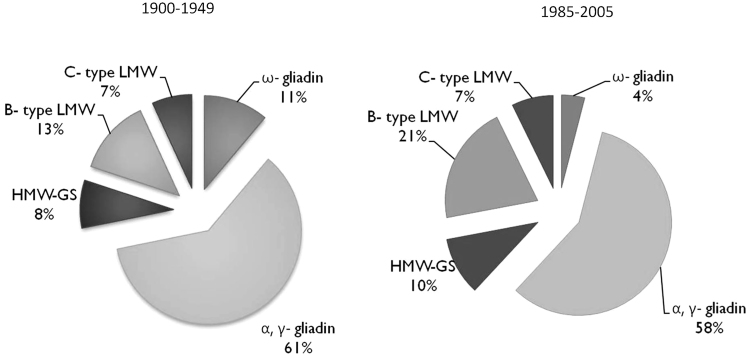
Storage protein composition in old (1900–1949) and modern (1985–2005) durum wheat groups normalised per protein unit. Values are means of two crop seasons.

After testing the variance homogeneity of the investigated parameters by Bartlett’s test, data were analysed using two different analysis of the variance (two-way ANOVA) considering as factors: (A) all genotypes and crop season and (B) the group of genotypes (1990–1949 and 1985–2005) and crop season as detailed in Table 2, Table 3. The significant differences among the mean values were calculated by Tukey’s test. Significant differences in storage protein composition observed between old and modern groups normalised for flour protein content (Fig. 3) were calculated by Student’s T-test. Due to the high correlations observed among the different variables, principal component analysis (PCA) was performed on the protein correlation matrix. The data set consisted of 90 samples tested with regard to nine variables. Before performing PCA, the values of each variable were standardised. The varimax method was chosen to obtain the best orthogonal factor rotation. The PCA results were graphically represented in two-dimensional plots. Statistical analysis was performed by software JMP (Version 8.0.2, SAS Institute Inc., 2009).

## 3 Results

### 3.1 Yield and quality parameters

Seven old (1900–1949) and eight modern (1985–2005) Italian durum wheat genotypes were grown under temperature conditions typical of the Mediterranean area, with lower temperatures during winter period and increasing temperature from spring. Rainfall distribution was not greatly different between the two crop seasons but there was a difference during grain filling with a slight water deficit in 2013. Analysis of variance showed high significant effects of genotype, crop season and their interactions for all the examined yield and quality parameters. In Table 2 grain yield, yield components and main technological indices are compared both within genotypes and between groups. In both crop seasons, as expected, the modern group showed significantly higher values with respect to the old one; among the old genotypes, Grifoni 235 showed the highest grain yield value, while among the modern genotypes Preco, Simeto and Adamello showed the lowest values. The modern genotypes generally showed higher grain yield in 2014 with significant differences for Claudio, Iride, Saragolla and PR22D89. Instead the old genotypes showed no significant differences in grain yield between the two crop seasons. The higher yield values observed in 2014 were consistent with a general increase in the number of kernels per unit area. As for the thousand kernel weights (TKW), significant differences between the two genotype groups were found only in the second crop season with the old genotypes showing the highest values. In the first crop season, among the old genotypes, Garigliano, Cappelli and Grifoni 235 showed the highest values and Timilia R.B. the lowest; instead among the modern genotypes, PR22D89 showed the highest value and Saragolla the lowest. On the contrary, in the second year, Saragolla showed the highest values in the modern group and Preco the lowest; among the old group, Timilia R.B., once again, showed the lowest value and Garigliano the highest.

**Table 2:** Grain yield and main quality parameters of all investigated genotypes (A) and of the two genotype groups (B) grown in 2013 and 2014.

	Genotype	Yield (t ha^−1^)		TKW (g)		kernel m^−2^ (number)		TW (kg hl^−1^)		GPC (% dm)		GI (%)	
		2013	2014	2013	2014	2013	2014	2013	2014	2013	2014	2013	2014
A	*1900–1949*												
	Dauno III	3.1^i−k^	3.1^i−k^	47.9^ef^	45.8^fg^	6389^j−m^	6842^h−m^	77.2^l^	78.4^k^	15.8^e^	14.2^i^	8^g^	7.3^g^
	old Saragolla	2.8 ^jk^	2.8 ^jk^	49.3^ef^	49.2^ef^	5765^k−m^	5791^k−m^	80.5^c^	76.6^m^	15.3^g^	16.3^c^	9^g^	5^g^
	Russello	2.9^i−k^	3.0^i−k^	48.8^ef^	54.8 ^bc^	6041^k−m^	5484^lm^	78.8^jk^	79.0^j^	16.6^b^	13.7^k^	7^g^	6^g^
	Timilia R.B.	3.7^f−i^	2.6^k^	36.0 ^jk^	34.7^j−l^	10246^ef^	7602^g−k^	80.4^h^	79.0^j^	16.5^b^	14.0^j^	5.2^g^	5.2^g^
	Cappelli	3.3^g−k^	2.8^g−k^	54.5^b−d^	48.5^ef^	6174^j−m^	5686^k−m^	81.1^c^	79.0^j^	16.9^a^	14.6^h^	9^g^	3^g^
	Garigliano	3.3^g−k^	3.4^g−k^	55.9 ^bc^	64.3^a^	5976^k−m^	5261^1m^	79.0^j^	76.0^n^	15.3^fg^	14.8^f^	6^g^	5.3^g^
	Grifoni 235	3.9^d−g^	3.4^f−i^	53.5^cd^	54.8^bc^	7381^g−m^	6292^j−m^	80.5^h^	75.5	15.8^e^	13.0^m^	13^g^	10.3^g^

	*1985–2005*												
	Adamello	3.9^e−h^	4.1^c−g^	55.4^bc^	37.5^j^	6990^g−m^	11044^de^	79.8^i^	67.4^s^	14.1^ij^	16.1^cd^	65^ab^	43^df^
	Simeto	3.9^d−h^	4.4^c−f^	47.5^ef^	38.0^ij^	8287^ghi^	11702^de^	79.7^I^	69.4^R^	15.5^f^	13.5^l^	46^df^	47^ce^
	Preco	3.7^f−i^	4.0^c−g^	57.3^b^	31.5^l^	6392^i−m^	12819^b−d^	79.7^ef^	66.9^t^	13.1^m^	16.0^de^	39^f^	13^g^
	Iride	4.6^c−e^	5.7^b^	54.2^bd^	35.9^jk^	8568^fgh^	15797^a^	82.3^d^	80.4^h^	13.1^m^	11.6^q^	48^cd^	45^df^
	Svevo	4.7^cd^	4.8^c^	45.8^fg^	33.6^kl^	10327^ef^	14176^ab^	82.1^de^	73.5^q^	16.2^cd^	15.1^g^	51^cd^	41^df^
	Claudio	4.5^c−e^	6.0^ab^	51.0^de^	41.5^hi^	8833^fg^	14358^ab^	84.3 ^a^	81.6^fg^	12.5°	11.8^q^	66^ab^	72^a^
	Saragolla	4.6^c−e^	6.6^a^	37.9^ij^	43.7 ^gh^	12086^cde^	15077 ^a^	82.9^c^	80.1^hi^	12.8^n^	12.1^p^	73^ab^	75^a^
	PR22D89	4.6^c−e^	5.7^b^	56.8^bc^	41.2 ^hi^	8091^g−j^	13928^a−c^	83.7^b^	74.6^p^	12.5 ^o^	13.0^m^	59^bc^	36^ef^

B	*1900–1949*	3.3^c^	3.0^c^	49.4 ^a^	50.3 ^a^	6853 ^c^	6137 ^c^	79.6 ^ab^	77.6 ^b^	16.0 ^a^	14.1 ^b^	9.6^b^	7.5^b^
	*1985–2005*	4.3^b^	5.2^a^	50.7 ^a^	37.9 ^b^	8697 ^b^	13613 ^a^	82.0 ^a^	74.2 ^c^	13.7 ^b^	13.6 ^b^	55.3^a^	46^a^

TKW = thousand kernel weight; TW = test weight; GPC = grain protein content; GI = gluten index. A = interaction genotype x crop season; B = interaction genotype group x crop season. For each parameter, values followed by different letters are significantly different at P ≤ 0.05 (small letters) and at P ≤ 0.001 (capital letters) according to the Tukey’s test.

Most of the genotypes showed significantly lower test weight (TW) in 2014 with only Dauno III and Simeto showing an opposite trend. A significantly higher grain protein content (GPC) was observed for the old genotypes in the first crop season (2013), while in 2014 no significant difference was found between the two genotype groups. A significant decrease in GPC occurred in 2014 only for the modern group, except for Adamello, Preco and PR22D89. The GI was significantly higher in the modern group of genotypes, the old genotypes showing very low values (minimum 5%, maximum 13%) without significant differences among genotypes and between the two crop seasons. In the modern group of durum wheat genotypes the GI ranged from 26 (Preco) to 74 (Saragolla). The effect of the crop season was significant only for three modern genotypes (Adamello, Preco, PR22D89), with higher values in the 2013 crop season.

### 3.2 Storage protein composition

Analysis of variance generally showed highly significant effects of genotype, crop season and of their interaction (GxY) on the gliadin to glutenin ratio (*glia:glut*) and on the relative proportions of all gliadin and glutenin subunits. However, no GxY effect on the proportion of ω-gliadins was observed. The full datasets for storage protein composition of the 15 genotypes in the two crop seasons are reported in Table 3. A comparison between the old and the modern groups was also performed on relative storage protein expression normalised per unit of semolina protein (Fig. 3).

**Table 3:** Storage protein composition of all investigated genotypes (A) and of the two genotype groups (B) grown in 2013 and 2014.

	Genotype	*glia:glut* (ratio)		HMW-GS (%)		B-type LMW-GS (%)		C- type LMW–GS (%)		HMW: B- LMW-GS (ratio)		ω − type gliadin (%)		α-, γ-type gliadin (%)	
		2013	2014	2013	2014	2013	2014	2013	2014	2013	2014	2013	2014	2013	2014
A	*1900–1949*														
	Dauno III	2.4^D−F^	2.9^C−E^	9.7^F^	9.2^F−H^	11.3^H−J^	8.1^J^	7.4^B−J^	7.3^B−J^	0.86^c^	1.13^b^	14.1^C^	11.2 ^DE^	56.3^D−K^	62.9^B−F^
	old Saragolla	1.8^D−F^	1.8^D−F^	9.3^FG^	7^MN^	19.4^C−F^	17.9^D−G^	4.3^E−K^	8.6^B−G^	0.48^g−m^	0.39^lm^	10.3^F^	10.8 ^EF^	54.5^F−L^	54.0^F−L^
	Russello	2.1^D−F^	1.8^D−F^	9.4^G−I^	9.7^G−I^	7.0^J^	13.3^G−I^	14.8^A^	10.8^A−C^	1.35^a^	0.72^c−f^	10.7^EF^	7.9^H^	55.1^E−L^	55.7^E−K^
	Timilia R.B.	3.3^B−D^	4.0^BC^	7.4^M^	5.6^Q^	8.8^IJ^	9.7^IJ^	5.7^D−K^	3.7^H−K^	0.84^c^	0.58^e−k^	18.2^A^	17.1^B^	58.5^C−J^	63.0^B−F^
	Cappelli	2.5^C−F^	2.6^C−F^	7.3^M^	6.1^PQ^	16.8^E−G^	16.7^E−G^	3.5^H−K^	4.4^E−K^	0.43^j−m^	0.37^lm^	9.2^G^	7.7^H^	62.0 ^B−G^	64.3^A−E^
	Garigliano	4.6^AB^	5.7^A^	6.2^OP^	4.9^R^	7.5^J^	6.64^J^	4.2^F−K^	2.7^JK^	0.83^cd^	0.74^c−e^	13.7 ^C^	11.7 ^D^	67.7^ABC^	73.4^A^
	Grifoni 235	1.5^EF^	1.7^D−F^	12.8^C^	8.6^JL^	17.3^E−G^	19.7^C−F^	9.2^B−F^	8.0^JK^	0.74^c−e^	0.44^j−m^	5.8^I^	4.8^JK^	52.9^G−L^	57.9^D−J^

	*1985–2005*														
	Adamello	1.9^D−F^	1.9^D−F^	8^L^	6.9^MN^	17.8^D−G^	14.7^F−H^	5.7^C−K^	10.7^A−C^	0.46^i−m^	0.47^h−m^	5.8^I^	4.5^KL^	60.3^B−H^	60.6^B−H^
	Simeto	2.0^D−F^	1.8^D−F^	8.5^IL^	9^G−J^	15.4^F−H^	16.7^E−G^	7.7^B−J^	7.8^B−J^	0.55^f−l^	0.54^g−l^	5.8^I^	5.2^IJ^	61.0^B−H^	59.1^C−I^
	Preco	2.1^D−F^	2.9^C−E^	9.3^F−H^	8.7^H−K^	19.7^C−F^	13.7^G−I^	1.8^K^	2.9^I−K^	0.47^h−m^	0.63^e−i^	5.1^JK^	4.6^KL^	62.9^B−F^	69.4^AB^
	Iride	1.3^EF^	2.2^D−F^	9.7^F^	7^MN^	23^BC^	17.7^D−G^	9.8^A−D^	6.7^B−K^	0.42^k−m^	0.40^k−m^	3.7^MN^	2.4^PQ^	52.1^H−L^	65.3^A−D^
	Svevo	1.4^EF^	1.0^F^	14.5^B^	15^A^	22.3^B−D^	25.9^B^	8.8^B−F^	7.2^B−J^	0.65^e−h^	0.58^e−k^	4.8^JK^	3.3^NO^	48.4^KL^	46.2^L^
	Claudio	1.4^EF^	1.4^EF^	11.3^D^	12.1^C^	17.3^E−G^	19.8^D−G^	11^AB^	9.4^B−E^	0.66^d−g^	0.61^e−j^	4.0^LM^	3.1^OP^	54.8^F−L^	54.7^F−L^
	Saragolla	1.1^F^	1.1^F^	10.5^E^	12.6^C^	31^A^	26.4^AB^	5.4^D−K^	7.6^B−J^	0.34^m^	0.48^g−m^	2.2^Q^	1.5^R^	49.7^J−L^	49.7^I−L^
	PR22D89	1.4^EF^	2.1^D−F^	9.3^F−H^	7^NO^	24.9^B^	19.2^C−F^	6.2^B−K^	4.9^D−K^	0.37^lm^	0.35^m^	3.7^MN^	3.5^M−O^	54.0^F−L^	64.3^A−E^

B	*1900–1949*	2.6^A^	2.9^A^	8.9^b^	7.3^c^	12.6^b^	13.2^b^	7.1^a^	6.5^a^	0.79^a^	0.63^ab^	11.7^a^	10.2^a^	58.1^AB^	61.6^A^
	*1985-2005*	1.6^B^	1.8^B^	10.1^a^	9.8^a^	21.4^a^	19.3^a^	7.1^a^	7.2^a^	0.49^b^	0.51^b^	4.4^b^	3.4^b^	55.4^B^	58.7^AB^

*glia: glut* = ratio of sum of gliadin subunits to sum of glutenin subunits; HMW: B- LMW-GS = ratio of expressed HMW-GS to B-type LMW-GS. A = interaction genotype x crop season; B = interaction genotype group x crop season. For each parameter, values followed by different letters are significantly different at P ≤ 0.05 (small letters) and at P ≤ 0.001 (capital letters) according to the Tukey’s test.

Significantly lower values for the *glia:glut* ratio were observed in the modern group of genotypes compared to the old ones (1.7 vs 2.8 in the mean of the years) in both crop seasons (Table 3). α, γ- gliadins were present in the highest proportion in all genotypes. Within the glutenins, the B-type LMW-GS were present in the highest proportion followed by HMW-GS and C-type LMW-GS.

#### 3.2.1 HMW-GS

All genotypes had the *Glu-A1* null allele, with the exception of Dauno III which appeared to have the *Glu-A1* HMW-GS 2 allele and *Glu-B1* HMW-GS 6 + 8 allele based on SDS-PAGE analysis (Table 1; Fig. 2a). The proportion of HMW-GS ranged from about 4.9% (Garigliano in 2014) to 15% (Svevo in 2014), as shown in Table 3, with expression being generally higher in the modern cultivars. Significant higher expression occurred in 2013 only in Grifoni 235. When the HMW-GS content was normalized per unit of flour protein (Fig. 3), a significantly higher expression was observed in the modern genotypes (8% vs 10%, P ≤ 0.001).

#### 3.2.2 B-type LMW-GS

Based on their mobility on SDS-PAGE (Fig. 2a) and the comparison with the literature (De Vita et al., 2007, Subira et al., 2014) it was concluded that all of the genotypes had the LMW-GS type 2 allele associated with the γ-45 gliadin, except for Russello which was characterised by a rare intralocus recombination resulting in the presence of the γ-45 gliadin and LMW-1 alleles, as reported by De Vita et al. (2007). Wide variation was observed in the proportion of B-type LMW-GS, ranging from 6.6% (Garigliano in 2014) to 31% (Saragolla in 2013) of the total storage proteins (Table 3). In general, the values were higher in the modern durum wheat genotypes than in the old genotypes in both years. Among the modern genotypes Preco, Iride and PR22D89 showed significantly higher values in 2013; among the old genotypes only the landrace Russello showed a significantly higher value in the 2013. When the proportion of B-type LMW-GS was normalized on semolina protein unit (Fig. 3) significantly higher values were observed in the modern genotypes, as shown in Fig. 3 (21% vs 13%; P ≤ 0.001).

#### 3.2.3 C-type LMW-GS

The patterns of LMW-GS below about 37 kDa differed between the genotypes (Fig. 2a), as reported previously (De Vita et al., 2007). The proportions of these bands varied widely among the old and modern groups of genotypes, ranging from 1.8% (Preco, in 2013) to 14.8% (Russello, in 2013) of the total gluten proteins (Table 3) but no differences between the two groups was observed. Similarly, no relationship with year of release was observed when the proportions of these bands were normalised on a flour protein basis (Fig. 3).

#### 3.2.4 HMW-GS: B-type LMW-GS ratio

Differences in the ratio of HMW-GS to B-type LMW-GS were found, as shown in Table 3. Analysis of variance showed a significantly higher ratio in the old genotypes than in modern ones only in the first crop season when, within the old group, Cappelli showed the lowest ratio, and the landraces Russello and Dauno III the highest. In the same year in the modern group the ratio ranged from 0.34 of Saragolla to 0.66 of Claudio. Significant differences between the two years were evident for Dauno III, Russello, Timilia R.B. and Grifoni 235 among the old genotypes and only for Preco among the modern genotypes. In general, higher ratios were observed in old genotypes in 2013, when water deficit occurred during grain filling, with the exception of Dauno III.

#### 3.2.5 ω–type gliadins

**Fig. 4:**
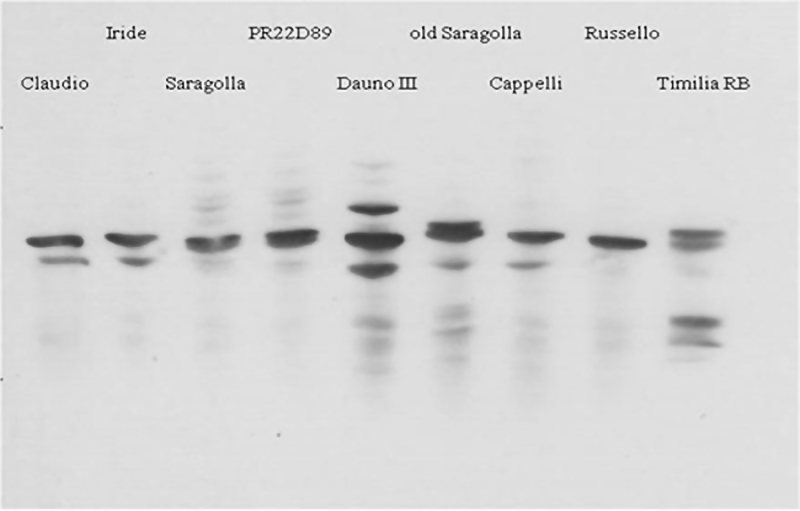
Western blot by monoclonal Ab specific to ω-5 gliadin (Tri a 19) in some old and modern genotypes.

**Fig. 5:**
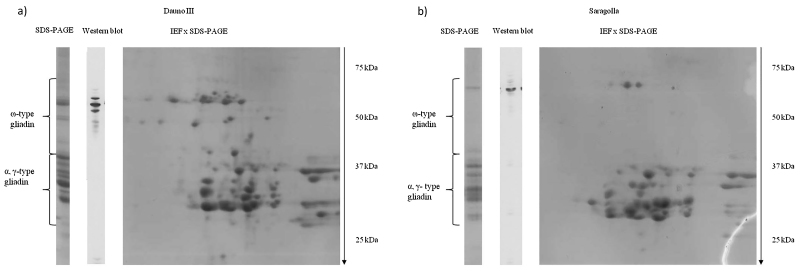
Analysis of gliadin composition by SDS-PAGE, western blot (ω-5 gliadin) and 2DE SDS-PAGE in old landrace Dauno III (a) and in modern cultivar Saragolla (b).

SDS-PAGE showed differences among the genotypes in the numbers of bands and their mobility in the 50–70 kDa region (Fig. 2b) indicating the presence of different allelic forms of ω–type gliadins, most of which had previously been genetically characterised (De Vita et al., 2007). Wide variation in the relative proportions of ω–gliadins (from 1.5 to 18.2% of the total storage proteins) was also observed (Table 3). In general, the proportion of ω–gliadins was higher in the old genotypes than in the modern ones in both years. The highest proportions of ω–gliadins were observed in the genotypes Timilia RB and Dauno III (18.2% in 2013, 17.1% in 2014, and 14.1% in 2013, 11.2% in 2014, respectively) and the lowest in modern Saragolla (2.2% and 1.5% in the first and second year, respectively). A trend toward a higher expression in the more stressed crop season (7.8% in 2013 vs 6.6% in 2014; P ≤ 0.001) was observed. Comparison of the ω–gliadin content of all genotypes, normalised per unit of protein in semolina (Fig. 3), showed clear genetic differences between the old and modern groups of durum wheat genotypes, with about three times lower expression in the modern group (4% vs 11%; P ≤ 0.001). Based on ω-gliadin polymorphism, five old (Dauno III, old Saragolla, Cappelli, Russello, Timilia RB) and four modern (Claudio, Iride, Saragolla and PR22D89) genotypes were analysed by western blot using a monoclonal ω-5 gliadin antibody (Fig. 4). The proportion of ω-5 gliadin bands expression on the total ω-type gliadins was about 75%, in a range of 50–80%. This proportion resulted significantly higher in 2013 (74% vs 67%; P ≤ 0.001), while no significant differences were found between the investigated old and modern groups of genotypes (71.4% vs 69.5%). On the basis of these results, Dauno III (old) and Saragolla (modern) were analysed in more detail by 2DE SDS-PAGE. This showed differences in the number and position of ω-gliadin spots between the two genotypes (Fig. 5). Dauno III showed five spots in the gel region corresponding to 55–58 kDa, with a pI from 5.4 to 7.2 pH. On the other hand, Saragolla showed three spots in the 60 kDa gel region with a narrow pI range (from 6.97 to 7.02). Dauno III also showed other six spots in 48–50 kDa gel region with a wide range of pI (5.0–7.8 pH), while no other spots were observed in ω-gliadin gel region for Saragolla.

#### 3.2.6 α-type and γ-type gliadins

The gel region from 30 to 43 kDa, corresponding to α-type and γ-type gliadins, accounted for the highest proportion of the total protein in all genotypes. The total proportion ranged from 46.2% (Svevo in the 2014) to 73.4% (Garigliano in 2014) of total storage proteins, as shown in Table 3. Several allelic forms were present, differing in the numbers of bands and their mobility (Fig. 2b). The mean values did not show significant differences either between the old and modern durum wheat groups, or between the two crop seasons; but the relative proportion of α-type and γ-type gliadins normalized per unit of semolina protein (Fig. 3) was higher in the old group (61% vs 58%; P ≤ 0.05).

### 3.3 Correlations and Principal Component Analysis

Correlations between GPC, GI and the storage protein composition are shown in Table 4. A highly significant positive correlation was found between GPC and the proportion of ω-type gliadins, and significant negative correlations between GPC and GI and B-type LMW-GS. The GI showed high significant positive correlations with the proportions of B-type LMW-GS and HMW-GS and high significant negative correlations with the *glia:glut* ratio, ω-type, α-type and γ-type gliadins and the HMW-GS: B-type LMW-GS ratio.

**Table 4:** Correlation matrix between the kernel quality parameters and gliadin and glutenin composition.

	GI	% ω- gliadin	% α-, γ- gliadin	% HMW-GS	% B- LMW-GS	% C- LMW-GS	HMW: B- LMW-GS	*glia: glut*
GPC	−0.53^**^	0.44^**^	0.00^ns^	−0.04^ns^	−0.33^*^	0.05^ns^	0.28^*^	0.11^ns^
GI		−0.75^**^	−0.41^**^	0.44^**^	0.70^**^	0.17^ns^	−0.42^**^	−0.60^**^
% ω- gliadin			0.32^*^	−0.48^**^	−0.75^**^	−0.22^ns^	0.49^**^	0.70^**^
% α, γ- gliadin				−0.81^**^	−0.65^**^	−0.56^**^	0.06^ns^	0.80^**^
% HMW-GS					0.55^**^	0.45^**^	0.14^ns^	−0.72^**^
% B- LMW-GS						0.02^ns^	−0.66^**^	−0.77^**^
% C- LMW-GS							0.34^*^	−0.52^**^
HMW: B-LMW-GS								0.32^*^

GPC = grain protein content; GI = gluten index; HMW: B-LMW-GS = ratio of expressed HMW-GS to B-type LMW-GS; *glia:glut* = ratio of sum of gliadin subunits to sum of glutenin subunits. n.s. = not significant; * = P ≤ 0.05; ** = P ≤ 0.001.

**Fig. 6:**
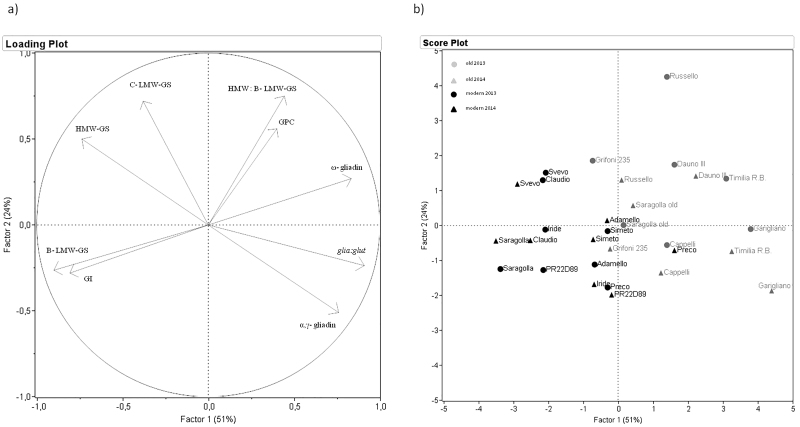
PCA loading plot showing the distribution of the analysed variables (a) and score plot (b) showing the distribution of old and modern genotypes grown in the two crop seasons along the two principal factors. GPC = grain protein content; GI = gluten index; glia:glut = ratio of sum of gliadin subunits to sum of glutenin subunits; HMW: B-LMW-GS = proportion of expressed HMW-GS to B-type LMW-GS ratio.

Principal component analysis (PCA) was performed on the correlation matrix. The first two factors explained 75% of the total observed variability (51% and 24% for Factor 1 and Factor 2, respectively), as reported in Table 5. The first factor was highly and positively associated with the gliadin subunits (ω–type and α-, γ-type) and the *glia:glut* ratio and negatively with the GI and both HMW and B-type LMW glutenin subunits. The second factor showed a positive association with the GPC*,* HMW-GS, C-type LMW-GS and the HMW-GS to B-type LMW-GS ratio, as shown in Fig. 6a. The distribution of the genotypes and of the two crop seasons along the two principal components is shown in Fig. 6b. A clear discrimination between the old and modern genotypes was observed along the Factor 1, except for Preco in 2014. On the basis of the PCA, the modern genotypes resulted associated with high GI and B-type LMW-GS, and with a low *glia:glut* ratio and low proportions of ω-type gliadins. The cultivar Saragolla showed the best technological quality which was associated with a high proportion of B-type LMW-GS and a low proportion of ω-type gliadins, although it also had a low protein content. The old group of genotypes showed greater variability in gluten protein composition in the two years, with the exception of genotype Cappelli that showed higher stability. Cultivar Grifoni 235, characterised by an intermediate year of release (1949), resulted distributed along the Factor 1 between the old and the modern genotypes.

**Table 5:** Varimax factor matrix.

	Factor 1	Factor 2
GPC	0.400	0.560
GI	−0.807	−0.279
*glia: glut*	0.909	−0.270
% ω- gliadin	0.834	0.270
% α, γ- gliadin	0.760	−0.508
% HMW-GS	−0.737	0.499
% B- LMW-GS	−0.901	−0.264
% C- LMW-GS	−0.380	0.721
HMW: B-LMW-GS	0.442	0.748
Eigenvalue	4.608	2.172
% of total variance	51%	24%

GPC = grain protein content; GI = gluten index; *glia:glut* = ratio of sum of gliadin subunits to sum of glutenin subunits; HMW: B-LMW-GS = proportion of expressed HMW-GS to B-type LMW-GS ratio.

## 4 Discussion

### 4.1 Breeding effect on yield and quality

The higher yield observed in the modern genotypes is a consequence of the primary target of 20th century breeding. An increase of 19.9 kg ha^−1^ year^−1^ was attributed to durum wheat genetic improvement by De Vita et al. (2007), who explained it by the shorter stature and greater earliness of the modern genotypes.

In the investigated genotypes the higher yield observed in the modern ones was related to the higher number of kernels per unit area. These results are in agreement with Subira et al. (2015) who compared Italian and Spanish durum wheat genotypes with different release dates, and with De Vita et al. (2007) who showed a significant correlation between the number of kernels per area unit and the year of release, in particular under low nitrogen input. Among the old genotypes, Timilia R.B. showed a different behaviour with the highest number of kernels per unit area and the lowest TKW; this result may be due to the “spring habitus” which characterizes this genotype determining more seeds with a low weight.

Significant differences between the two crop seasons were mainly found for the modern genotypes, while the old ones showed a greater yield stability. This result might be also due to the different heading time, being the modern genotypes earlier than the old ones. Indeed, in 2013 characterised by a slight water stress during grain filling, the heading time for modern genotypes was in late April (Table 1) when water deficit occurred (Fig. 1), whereas for the old genotypes heading occurred on May, when water was not a limiting factor.

The old genotypes had higher GPC, in agreement with De Vita et al. (2007) and resulting from the negative correlation (−0.53, P ≤ 0.001; data not shown) between yield and protein content (Giuliani et al., 2011, Toscano et al., 2014). Among the modern genotypes, the cultivar Svevo showed high GPC in both crop seasons; this genotype has recently been characterised as having the Fd-GOGAT gene on chromosome 2A, which is a major QTL for GPC in durum wheat (Nigro et al., 2014). By contrast, two old (Timilia R.B. and Grifoni 235) and three modern (Adamello, Preco and PR22D89) genotypes consistently showed a higher protein content associated with higher yield and TKW. In this case, better environmental conditions may have resulted in both higher yield and better nitrogen assimilation. The GI, a widely accepted indicator of gluten strength, was reported to be more influenced by the genotype than by the crop season by Sissons (2008) and Flagella et al. (2010). A marked improvement in gluten strength was also observed in the modern genotypes which showed a mean GI value of 50% vs 8.5% of the old genotypes under the low nitrogen input applied. This result confirmed the increase in pasta-making quality during the 20th century in durum wheat cultivars reported by De Vita et al. (2007) for Italian cultivars and by Subira et al. (2014) who found increases in gluten strength of 32.1% (0.54% year^−1^) and 27.9% (0.33% year^−1^) in Italian and Spanish cultivars, respectively. This improvement in gluten strength was attributed to the incorporation of favorable alleles in modern cultivars, such as the 7 + 8 HMW-GS encoded by the *Glu*-*B1* locus (De Vita et al., 2007) and to the increased frequency of the LMW-GS combination *aaa*, which was present in 75% of all intermediate cultivars and in 100% of the modern Italian cultivars (Subira et al., 2014).

### 4.2 Breeding effect on gluten composition in relation to technological and healthy quality

The Italian durum wheat genotypes used in our study were representative of two release periods (*1900–1949* and *1985–2005*) and of the most frequent allelic forms of HMW-GS in Mediterranean durum wheat genotypes: *Glu-B1* 20, 7 + 8 and 6 + 8 (Subira et al., 2014). Most studies have focused on allelic composition (De Vita et al., 2007, Subira et al., 2014) and do not provide a complete explanation for differences in gluten quality (GI) among genotypes with different release dates. In particular, differences in gluten strength between genotypes with the same allelic composition could be explained by differences in storage protein subunit amount.

#### 4.2.1 Glutenin composition

A lower *glia:glut* ratio was observed in the modern cultivars than in the old ones which was associated with an improvement in the gluten index. This result is in accordance with Sissons et al. (2005) who found that the mixograph dough strength increased with a higher proportion of glutenins on gliadins, even when studying glutenin-enriched flours.

The impact of the LMW-2 has been widely studied. Several studies on allelic LMW composition and dough strength (D’Ovidio and Masci, 2004, Sissons et al., 2005) demonstrated the role of LMW-2. Edwards et al. (2007) found an association between gluten strength and LMW-2. Masci et al. (2000) also found a strong correlation between the 42 kDa LMW band and gluten strength, with the major protein in the band being identified as LMW-m type (D’Ovidio and Masci, 2004, Muccilli et al., 2010, Giuliani et al., 2015). Gluten quality, measured as GI, was most greatly affected by the relative expression of B-type LMW-GS (R = 0.7; P ≤ 0.001). This effect was more evident in the modern cultivars, characterised by the 7 + 8/6 + 8 HMW *Glu-B1* alleles. The greater expression of B-type LMW-GS resulting from breeding was therefore associated with improvement of the GI. Among the genotypes studied, Saragolla showed the highest GI (>75%) and the highest expression of B-type LMW-GS (>26% of total storage proteins). This association seems confirmed also for Preco, that showed in 2014 the consistent lowest GI and B-type LMW-GS expression. It is probable that the selection of durum wheat for higher pasta-making quality (GI, alveograph and mixograph properties; De Vita et al., 2007) resulted in the selection of genotypes with greater relative expression of B-type LMW-GS, in particular the 42 kDa band. This protein plays a key role in dough resistance and elasticity, and it is characterised by the presence of two cysteines available for inter-chain disulphide bond formation (D’Ovidio and Masci, 2004). Masci et al. (2000) reported that the weak dough strength of the durum wheat cultivar Demetra probably resulted from low expression of LMW-2 allele. Subira et al. (2014) found a higher frequency of the LMW-GS *aaa* allelic combination in intermediate and modern Italian and Spanish varieties, which was associated with higher gluten strength. The proportion of C-type LMW-GS, which has been proposed to act as chain terminators (D’Ovidio and Masci, 2004) in the gluten network, was not significantly correlated with the year of release. The HMW-GS showed a slight increase in relative expression in the modern genotypes. HMW-GS in general account for about 9–11% of total storage proteins (Shewry, 2009). The role of the HMW-GS (Shewry, 2009) and of their allelic forms (De Vita et al., 2007, Sissons, 2008, Subira et al., 2014) on gluten technological properties is known. The ratio between HMW-GS and B-type LMW-GS significantly decreased with the year of release; this correlation resulted mainly from the increased expression of B-type LMW-GS in the modern varieties.

#### 4.2.2 Gliadin composition and ω-5 gliadin expression

A markedly higher proportion of ω-type gliadins was observed in the old genotypes. Since the proportion of ω-gliadins showed a significant correlation with grain protein content, the relative proportion of this gliadin sub-fraction was also calculated after normalization per unit of protein. Even after this normalization, the average ω-gliadin expression level was about three times higher in the old genotypes. Among the modern genotypes, Saragolla showed the best gluten quality composition with the lowest proportion of ω-gliadins and the highest of B-type LMW-GS. These results are in agreement with Wieser et al. (1994) who observed a range of contents of ω-gliadins in bread wheat varieties, from 6.2 to 20% of the total gliadins. The S-poor ω-gliadins do not have a direct impact on pasta quality (Sissons, 2008); on the other hand, ω-5 gliadins are of particular interest because of their role in wheat allergy (Matsuo et al., 2015). In particular, ω-5 gliadins are major components responsible for triggering wheat-dependent exercise-induced anaphylaxis (WDEIA). On the basis of the western blot and 2D SDS-PAGE, the high expression of ω-type gliadins observed in the old landrace Dauno III and, by contrast, the low expression in the modern cultivar Saragolla, resulted from differences in the numbers of bands on SDS-PAGE (higher in Dauno III) and isoforms on 2DE. Also Denery-Papini et al. (2007) previously observed a variability in ω-gliadin polymorphism. In particular, the authors found varietal differences in the proportions of ω-5 gliadins allelic forms in bread wheat, associated to differences in reactivity to specific ω-5 antibody and IgE patient sera. The differences in ω-gliadin expression observed between the old and the modern groups of durum wheat genotypes are of great interest in relation to the potential allergenicity of pasta wheat. In a recent study with transgenic bread wheat in which ω-5 gliadin genes were silenced by RNA interference (Altenbach et al., 2015), reduced binding was observed with sera from allergic patients. It may therefore be possible to exploit variation in wheat germplasm to produce cultivars with reduced amounts of ω-5 gliadins. It is known that ω-gliadin expression is highly affected by the environment (Hurkman et al., 2013, Wan et al., 2014, Altenbach et al., 2015). Our study showed that ω-gliadin expression was generally higher in 2013, also characterised by lower rainfall during grain filling. In literature an increase in ω-gliadin under water stress was already observed by Hurkman et al. (2013) and Giuliani et al. (2015).

It is known that α-type and γ-type gliadins contain many T-cell epitopes for coeliac disease (Sollid et al., 2012). In the present study no significant effects of breeding were found for α-type and γ-type gliadin expression. Moreover, Ribeiro et al. (2016) did not find a correlation between α-gliadin content and the CD toxicity of gluten which was estimated using the R5 competitive ELISA immunoassay but did find a relationship with the ω-gliadin content in tetraploid wheats. However, the authors stated that further studies are necessary to support this result. van den Broeck et al. (2010) reported that modern bread wheat cultivars tended to show higher contents of the major Glia-α9 coeliac disease epitope and a lower content of the minor Glia-α20 epitope; however, lines showing high and low reactions with both antibodies were present in both sets of germplasm. Recently Ribeiro et al. (2016) reported that breeding did not appear to have contributed to an increase in the prevalence of coeliac disease epitopes.

## 5 Conclusions

In this paper differences in gluten composition between old and modern groups of durum wheat genotypes were evaluated in relation to durum wheat breeding in the 20th century in Italy. This phenotyping was performed under the Mediterranean environment, where water availability and thermal stress during grain filling are key factors responsible for agronomic performance, yield and quality.

Relative to the differences between the two crop seasons, the proportions of ω-gliadins and HMW-GS were generally higher when rainfall during grain filling was lower.

Comparisons of the two groups of genotypes showed that the better technological performance of modern varieties has not only resulted from improved glutenin allelic composition due to the introduction of high quality alleles at the *Glu-B1* and *Glu-B3* loci, but also due to differential expression of specific storage proteins. In particular, the higher gluten index observed in modern genotypes was correlated with an increased content of glutenins, especially B-type LMW-GS. No significant differences were found between old and modern durum wheat genotypes in relation to α-type and γ-type gliadins, the former being considered one of the major fractions determining coeliac disease toxicity. Furthermore, a drastic decrease in the expression of ω-type gliadin, mainly represented by ω-5 gliadin (also known as Tri a 19) which is the major allergen in WDEIA, was observed in the modern genotypes.

In conclusion, durum wheat breeding carried out in Italy during 20th century seems to have improved wheat gluten quality in relation to both technological performance and allergenic potential. In particular, the introduction of high quality alleles and the higher expression of B-type LMW-GS are responsible for better gluten strength and the marked decrease in ω-type and in particular ω-5 gliadin expression may indicate a potential lower allergenicity of modern varieties. Further studies are needed to explore this topic and to support these results, also in relation to environmental and management influence, by applying the new −omics technologies.
